# Awareness and Perception Toward Nicotine Replacement Therapy Among Medical Students at Umm Al-Qura University

**DOI:** 10.7759/cureus.44343

**Published:** 2023-08-29

**Authors:** Safwan Almehmadi, Mariah Almehmadi, Fatima Alhajaji, Fahad Alharbi, Salihah Alqorashi, Muath Alqahtani, Yosra Z Alhindi

**Affiliations:** 1 Medicine and Surgery, Umm Al-Qura University, Makkah, SAU; 2 Pharmacology and Toxicology, Umm Al-Qura University, Faculty of Medicine, Makkah, SAU

**Keywords:** umm al-qura university, medical students, smoking cessation, cigarrete, nicotine replacment therapy, nicotine

## Abstract

Background

Tobacco smoking is a leading cause of premature mortality worldwide, with most deaths attributed to smoking-related diseases. Quitting smoking can have significant health benefits and increase one’s lifespan. Nicotine, the addictive component of tobacco, can lead to cravings and withdrawal symptoms. Nicotine replacement therapy (NRT) can be an effective aid in easing these symptoms. This study aimed to estimate the awareness, acceptance, knowledge, and perception of NRT among medical students at Umm Al-Qura University in Makkah City.

Methodology

A cross-sectional survey method using Google Forms was utilized to distribute a questionnaire among medical students. The data were analyzed data using RStudio with statistical significance set at p-values <0.05.

Results

Of the 310 students included in this study, the majority were males (56.5%) and were aged between 21 and 24 years. Overall, 31.9% of the participants were in their fourth year of study. Regression analysis showed that being in the fifth or sixth academic year significantly predicted awareness of NRT.

Conclusions

Senior-year medical students had more knowledge and awareness about NRT than their junior colleagues. Future recommendations are vital for medical students to increase their knowledge, awareness, and practice regarding NRT.

## Introduction

Globally, smoking is a significant cause of early mortality [1,2]. Many of the excess deaths among smokers are caused by smoking-related neoplastic, vascular, pulmonary, and other illnesses. Despite using tobacco for long periods of time in various ways, quitting smoking can significantly improve the general health of patients. When compared to people who had never smoked, the lifespan of tobacco users was reduced by more than 10 years. Adults who had quit smoking between the ages of 25 and 54 years earned 6-10 years of life compared to those who had not [3].

Nicotine is the main active component of tobacco and is the primary factor controlling the psychopharmacological effects related to addiction [4]. Through the administration of nicotine, nicotine replacement treatment (NRT) aims to decrease the desire to use cigarettes and withdrawal symptoms [5]. Evidence shows that NRT aids in quitting smoking and has become widely accepted. Various healthcare professionals advise using NRT as the first-line therapy for smokers who seek pharmacological help [6]. In the absence of constraints, at least one of the five first-line pharmacotherapies for NRT such as nicotine gum, nicotine inhaler, nicotine nasal spray, and nicotine patch should be given. All health insurance plans should include as a reimbursed benefit the counseling and the above-mentioned pharmacotherapeutic treatments as successful in the updated recommendation because NRT is cost-effective in comparison to other medical and disease prevention initiatives [7].

A study examined the knowledge and perceptions of NRT among dental professionals in Riyadh City, Kingdom of Saudi Arabia. Almost 74.4% of the participants knew about NRT, and 50% of students believed that motivating about NRT is a waste of time. About 65.2% of students thought it was difficult to stop smoking, and 72.7% believed that NRT plays a big role in helping people quit smoking [8].

This study aimed to examine the awareness, acceptance, knowledge, and perception of NRT among medical students at Umm Al-Qura University (UQU) in Makkah City. The primary objective was to assess the level of knowledge and perception among medical students in Makkah toward NRT. The secondary objectives were to assess medical students’ awareness regarding smoking cessation by NRT, determine the prevalence of NRT use among medical students in UQU, explore the relationship between the level of NRT knowledge and different sociodemographic characteristics, and explore the most common sources of medical information among medical students in Makkah City.

## Materials and methods

Study design

This retrospective, cross-sectional study was conducted at UQU in the western region of Saudi Arabia from January to July 2023. Data were obtained using an online questionnaire directed to medical students at UQU.

Inclusion and exclusion criteria

The selection criteria included all males and females in the preclinical and clinical years from the second to the sixth years irrespective of whether they were smokers or non-smokers. Other healthcare students, such as pharmacists and nurses, were excluded.

Sample size determination

OpenEpi version 3.0 was used to determine the minimal sample size needed for this study, keeping the confidence interval (CI) level at 95%, assuming a 50% prevalence of knowledge [7] about NRT, and the overall population of about 1300 students. According to calculations, a total of 297 participants accounted for the sample. We increased the sample size to 310 participants to account for possible data loss and decrease bias.

Study procedures

To identify the target population, we contacted academic affairs to provide the list of all medical students. The estimated number was 1300 full-time undergraduate medical students at UQU Medical College who were contacted to participate in the survey.

Our target was to design a survey that could be readily comprehended by medical students. Questionnaire items were determined based on a literature review. We divided items into the following categories: sociodemographic information, academic performance, awareness of NRT, acceptance of NRT, application of NRT, and personal experiences with NRT.

The survey was distributed in the English language and was developed using Google Forms. We used the validation method and administered the survey to 10 medical students from another university.

We send electronic messages accompanied by questionnaire objectives, the target population, and a request for voluntary participation. All students who met the inclusion criteria received the survey electronically via social media applications. To ensure the privacy of participant information, a system of codes, numbers, and pseudonyms was established. The data were only available to researchers.

Ethical approval

The study was approved by the Biomedical Research Ethics Committee, Faculty of Medicine, Umm Al-Qura University, Makkah, Saudi Arabia (approval number: HAPO-02-K-012-2023-04-1591). The study followed the guidelines of the Declaration of Helsinki.

Statistical analysis

The statistical analysis was performed using RStudio (R version 4.3.0). The demographic data as well as knowledge and awareness about NRT were presented as frequencies and percentages. The chi-square test was used to investigate the association between the demographic data and the awareness and practice toward NRT.

Multiple logistic regression was used to determine the predicting factors such as participants’ gender, age, academic years, GPA, and awareness toward NRT. We used odds ratios (ORs) and 95% CIs to present the results of the regression analysis. Statistical significance was considered at p-values <0.05.

## Results

Demographic and academic characteristics

We analyzed the data of 310 students. Males accounted for most of the participants (175, 56.5%), and most of the participants were aged 21 to 24 years. About 99 (31.9%) participants were fourth-year medical students. Approximately two-thirds of the students had a GPA of 3.5 to 4.0 (201, 64.8%) (Table 1).

**Table 1:** Demographic and academic characteristics.

Parameter	Category	N (%)
Gender	Male	175 (56.5%)
	Female	135 (43.5%)
Age groups (year)	18–20	61 (19.7%)
	21–24	233 (75.2%)
	25–32	16 (5.2%)
Academic year	Second-year medical school	45 (14.5%)
	Third-year medical school	66 (21.3%)
	Fourth-year medical school	99 (31.9%)
	Fifth-year medical school	25 (8.1%)
	Sixth-year medical school	75 (24.2%)
GPA	<2.0	1 (0.3%)
	2.0 to <2.5	1 (0.3%)
	2.5 to <3.0	15 (4.8%)
	3.0 to <3.5	92 (29.7%)
	3.5 to 4.0	201 (64.8%)

Awareness regarding nicotine replacement therapy

In general, 188 students were aware of NRT, representing 60.6% of the sample. Awareness regarding NRT was significantly higher among males (69.7% among males vs. 48.9% among females; p < 0.001), students aged 25 to 32 years (87.5% among those aged 25-32 years vs. 63.1% among those aged 21-24 years vs. 44.3% among those aged 18-20 years; p = 0.002), students in the fifth and sixth academic years (88.0% and 90.7%, respectively) compared to those in the fourth (48.5%), third (47.0%), and second years (42.2%) (p < 0.001). Furthermore, the proportion of aware students was significantly higher among those who had a GPA of 3.0 to <3.5 (72.8%) compared to those with a GPA of 3.5 to 4.0 (55.7%), 2.5 to <3.0 (53.3%) and <2.5 (50.0%) (p = 0.021) (Table 2). On regression analysis, awareness regarding NRT was significantly high among students in the fifth (OR = 9.2, 95% CI = 2.2 to 49.1, p = 0.004) and sixth academic years (OR = 11.2, 95% CI = 3.3 to 41.6, p < 0.0001 (Table 3).

**Table 2:** Awareness of nicotine replacement therapy. NRT: nicotine replacement therapy

Parameter	Category	Have you ever heard about NRT?				P-value
		No		Yes		
		N	%	N	%	
Gender	Male	53	30.30%	122	69.70%	<0.001
	Female	69	51.10%	66	48.90%	
Age groups (year)	18–20	34	55.70%	27	44.30%	0.002
	21–24	86	36.90%	147	63.10%	
	25–32	2	12.50%	14	87.50%	
Academic year	Second-year medical school	26	57.80%	19	42.20%	<0.001
	Third-year medical school	35	53.00%	31	47.00%	
	Fourth-year medical school	51	51.50%	48	48.50%	
	Fifth-year medical school	3	12.00%	22	88.00%	
	Sixth-year medical school	7	9.30%	68	90.70%	
GPA	<2.5	1	50.00%	1	50.00%	0.021
	2.5 to <3.0	7	46.70%	8	53.30%	
	3.0 to <3.5	25	27.20%	67	72.80%	
	3.5 to 4.0	89	44.30%	112	55.70%	

**Table 3:** Regression analysis of awareness about nicotine replacement therapy.

Parameter	Category	OR	95% CI	P-value
Gender	Male	Ref	Ref	
	Female	0.77	0.44, 1.37	0.378
Age groups (year)	18–20	Ref	Ref	
	21–24	0.85	0.36, 1.95	0.693
	25–32	1.03	0.16, 11.5	0.975
Academic year	Second-year medical school	Ref	Ref	
	Third-year medical school	1.13	0.46, 2.77	0.786
	Fourth-year medical school	1.3	0.49, 3.45	0.598
	Fifth-year medical school	9.15	2.20, 49.1	0.004
	Sixth-year medical school	11.2	3.31, 41.6	<0.001
GPA of the last year	<2.5	Ref	Ref	
	2.5 to <3.0	1.98	0.04, 80.1	0.702
	3.0 to <3.5	4.44	0.11, 159	0.385
	3.5 to 4.0	2.78	0.07, 98.2	0.549

Perceptions and attitudes toward nicotine replacement therapy

Large proportions of students indicated that smoking cessation includes NRT (79.0%) and that NRT can be availed in the form of nicotine gums, nicotine patches, and nicotine nasal spray (72.9%). Moreover, many students thought that undergraduate students have a role in tobacco cessation (78.7%), young age is a motivating factor for tobacco cessation via NRT (71.9%), it is difficult to quit (71.6%), and that motivating is not a waste of time (82.9%) (Table 4). Notably, the most common source of information about NRT was the internet (84.5%), whereas other sources included newsletters and books (8.1%) and TV or radio.

**Table 4:** Perceptions and attitudes toward nicotine replacement therapy. NRT: nicotine replacement therapy

Parameter	Category	N (%)
Does smoking cessation include NRT?	No	65 (21.0%)
	Yes	245 (79.0%)
NRT can be availed in which of the following forms:	Nicotine gum	18 (5.8%)
	Nicotine patches	64 (20.6%)
	Nicotine nasal spray	2 (0.6%)
	All of the above	226 (72.9%)
Do you think undergraduate medical students have a role in tobacco cessation?	No	66 (21.3%)
	Yes	244 (78.7%)
Does young age motivate others for tobacco cessation by talking about NRT?	No	87 (28.1%)
	Yes	223 (71.9%)
Do you think motivating is a waste of time?	No	257 (82.9%)
	Yes	53 (17.1%)
Is it a difficult task to quit?	No	88 (28.4%)
	Yes	222 (71.6%)
From where do you get your information?	Internet	150 (84.5%)
	Newsletters and books	25 (8.1%)
	TV	23 (7.4%)

Previous experience with nicotine replacement therapy

Only a small percentage of students (7.4%) had tried NRT. There were no significant differences in the practice of NRT regarding students’ gender, age groups, academic year groups, and GPA (Table 5).

**Table 5:** Previous experience with nicotine replacement therapy. NRT: nicotine replacement therapy

Parameter	Category	Have you ever tried NRT?				P-value
		No		Yes		
		N	%	N	%	
Gender	Male	158	90.30%	17	9.70%	0.079
	Female	129	95.60%	6	4.40%	
Age groups (year)	18–20	54	88.50%	7	11.50%	0.347
	21–24	218	93.60%	15	6.40%	
	25–32	15	93.80%	1	6.30%	
Academic year	Second-year medical school	41	91.10%	4	8.90%	0.958
	Third-year medical school	62	93.90%	4	6.10%	
	Fourth-year medical school	91	91.90%	8	8.10%	
	Fifth-year medical school	24	96.00%	1	4.00%	
	Sixth-year medical school	69	92.00%	6	8.00%	
GPA	<2.5	1	50.00%	1	50.00%	0.239
	2.5 to <3.0	14	93.30%	1	6.70%	
	3.0 to <3.5	85	92.40%	7	7.60%	
	3.5 to 4.0	187	93.00%	14	7.00%	

## Discussion

Nicotine addiction is a complicated issue that has biological, psychological, and behavioral implications, which can lead to many premature disabilities and deaths [9]. Smoking nicotine not only affects general health and causes pathologies to an individual it also carries financial implications for the family and depreciates the quality of life. Smokers spend a large amount of money on their addiction to smoking. Because of the history of failing to abstain from nicotine, individuals generally give up and become disappointed. This leads to the impossible task of achieving and tolerating withdrawal symptoms [8]. Nicotine addiction is the most significant factor in quitting smoking and maintaining continued abstinence [10].

NRT is the most common form of pharmacotherapy for smoking cessation which is effective in treating tobacco dependence. NRT aims to temporarily replace most of the nicotine found in cigarettes to reduce the desire to smoke and the symptoms of nicotine withdrawal symptoms, thereby facilitating the transition from cigarette smoking to complete abstinence [11].

Nowadays, various nicotine replacement modalities are offered in various dosages, tastes, and forms, and their usage is advised for all smokers who do not have a medical condition that precludes it. The patient’s choices should typically be taken into consideration while selecting an NRT product [12].

Several options are available for treating tobacco addiction with proven effectiveness, including behavioral and pharmacological therapies. These therapies vary in their efficacy, acceptability, and cost-effectiveness [13].

A few of the factors that have been identified as causing low NRT compliance [14] include concerns related to safety [15], addiction to NRT, adverse reactions, cost, and relapse [16,17]. The effectiveness of NRT is one of the most significant causes of low compliance. Patients may believe that the treatment is no longer required when desire and withdrawal are well managed with medication [18]. This can be prevented by health practitioners providing NRT-related scientific knowledge to individuals [19].

In this study, medical students at UQU were assessed about their general awareness of NRT. Overall, 60.6% of medical students were aware of NRT while the rest were unaware. Around 69.7% of aware students were male and 48.9% were female. The most aware age groups were 25-32 years (87.5%). Sixth-year medical students were most aware of NRT (90.7%). Overall, 72.8% of students with a GPA of 3.0 to <3.5 were aware, which represents a positive relationship between the awareness of NRT and GPA. This can be explained based on growing through education as fifth and sixth-year students would have had more knowledge, information, and awareness than their junior colleagues.

Moreover, around 79% agreed that smoking cessation should include NRT while the rest disagreed. About 5.8% knew about gum patches, 20.6% knew about nicotine patches, 0.6% knew about internasal sprays, and 72.9% believed that NRT is available in all the above forms.

Furthermore, the study showed that 78.7% of undergraduate medical students had a role in tobacco cessation and 71.9% approved that young age motivates others for tobacco cessation through community awareness about NRT. Fortunately, 82.9% thought that motivation is not a waste of time, but 71.6% accepted that quitting smoking is a difficult task. Most students sought information from the Internet (84.5%).

In this research, 9.7% of males had tried NRT while 4.4% of females had tried NRT. The age group that had tried NRT the most was 18-20 years old at 11.5%, and the academic year most associated with NRT use was the second year at 8.9%.

A similar study investigating the awareness of NRT among dental students found that despite having a positive attitude toward NRT, only 34% of the postgraduate students and 57% of the faculty members claimed to practice NRT along with tobacco cessation counseling [16].

In a study investigating the prevalence of e-cigarette use among medical students in UQU, e-cigarette use was relatively high and their knowledge was insufficient.

Previous studies have shown that NRT is a successful intervention for smokers who refuse to or cannot attempt an abrupt quit and want to stop smoking for the long term [20]. A meta-analysis concluded that NRT improves the chances of habit reduction among smokers who are not ready to quit smoking entirely [21]. Young individuals usually have misconceptions about nicotine. To maximize the positive effects on public health, following the FDA’s proposed nicotine reduction rules, mandatory nicotine warning labels, and public education are essential [22,23].

One of this present study’s limitations is that it involved only medical students from UQU. For more comprehensive findings, opinions and knowledge of other students also need to be assessed such as dental students, pharmacists, and nurse students As this is a cross-sectional study assessing exposure and outcomes for the first time, it is difficult to derive causal relationships. Moreover, these studies are also prone to certain biases.

## Conclusions

In this study, 310 UQU medical students were assessed about their general awareness of NRT. Males accounted for most of the study population aged 21 to 24 years old. Overall, 60.6% of medical students were aware of NRT, and, fortunately, 82.9% believed that motivation is not a waste of time. Fifth and sixth-year medical students’ awareness of NRT was significantly higher than that of other students. Future studies are vital for medical students to increase their knowledge and awareness regarding the practice of NRT through health educational programs. Furthermore, it is important to determine how practicing physicians and postgraduate students assess, accept, use, and apply NRT.
